# Thin-Reinforced Anion-Exchange Membranes with High Ionic Contents for Electrochemical Energy Conversion Processes

**DOI:** 10.3390/membranes12020196

**Published:** 2022-02-08

**Authors:** Hyeon-Bee Song, Do-Hyeong Kim, Moon-Sung Kang

**Affiliations:** Department of Green Chemical Engineering, College of Engineering, Sangmyung University, Cheonan 31066, Korea; gusql1231@gmail.com (H.-B.S.); dohyeongkim665@gmail.com (D.-H.K.)

**Keywords:** ion-exchange membranes, electrochemical energy conversion, reverse electrodialysis, all-vanadium redox flow battery, porous substrate, degree of swelling, double cross-linking

## Abstract

Ion-exchange membranes (IEMs) are a core component that greatly affects the performance of electrochemical energy conversion processes such as reverse electrodialysis (RED) and all-vanadium redox flow battery (VRFB). The IEMs used in electrochemical energy conversion processes require low mass transfer resistance, high permselectivity, excellent durability, and also need to be inexpensive to manufacture. Therefore, in this study, thin-reinforced anion-exchange membranes with excellent physical and chemical stabilities were developed by filling a polyethylene porous substrate with functional monomers, and through in situ polymerization and post-treatments. In particular, the thin-reinforced membranes were made to have a high ion-exchange capacity and a limited degree of swelling at the same time through a double cross-linking reaction. The prepared membranes were shown to possess both strong tensile strength (>120 MPa) and low electrical resistance (<1 Ohm cm^2^). As a result of applying them to RED and VRFB, the performances were shown to be superior to those of the commercial membrane (AMX, Astom Corp., Japan) in the optimal composition. In addition, the prepared membranes were found to have high oxidation stability, enough for practical applications.

## 1. Introduction

**Figure 1:**
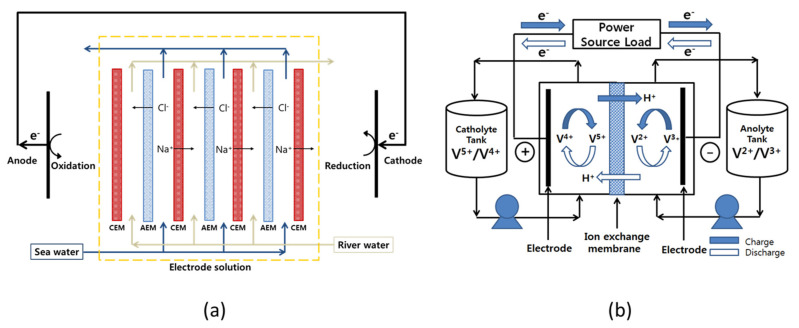
Schematic diagram showing the configuration and working principle of (**a**) reverse electrodialysis and (**b**) all-vanadium redox flow battery.

Recently, as global warming and climate change are accelerating, interest in eco-friendly renewable energy technologies with little or no carbon emission is increasing worldwide [1,2]. Among various eco-friendly renewable energy technologies, research on salinity gradient power generation using the concentration difference between seawater and freshwater is being actively conducted. Compared to other competitive energy technologies, such as solar power and wind power, salinity gradient power generation has the advantage of being less affected by the surrounding environment and not having severe power fluctuations. Representative salinity gradient power generation technologies using membranes include pressure-retarded osmosis (PRO) and reverse electrodialysis (RED). Between them, PRO is a method of producing electricity by converting the kinetic energy generated by the salinity difference [3,4]. Therefore, it requires a turbine and high-pressure equipment and also has problems such as deterioration of the performances of the semi-permeable membranes owing to severe concentration polarization. On the other hand, RED employs ion-exchange membranes (IEMs) to selectively transport ions using the concentration difference between seawater and freshwater as a driving force and generates electricity through oxidation-reduction reactions at the electrodes. In this case, it is known to be more efficient than PRO power generation because chemical energy is directly converted into electricity [4,5]. Figure 1a displays the schematic diagram showing the operating principle of RED.

The IEM is one of the core components that determine the power generation performance of RED. The open-circuit voltage (OCV) shown in equation (1) is a performance parameter that greatly affects the power generation efficiency of RED:

(1) $$V^{o}=N\frac{2\alpha RT}{zF}\ln\left(\frac{a_{c}}{a_{d}}\right)$$

where *V^o^* is the OCV, *N* is the number of the IEMs, *α* is the average permselectivity of the IEMs, *R* is the ideal gas constant (8.314 J/mol K), *T* is the absolute temperature (K), *z* is the valency, *F* is the Faraday constant (96,485 C/mol), and *a_c_* and *a_d_* are the concentrations (mol/L, M) of seawater and freshwater, respectively. From Equation (1), it can be seen that the *α* value, which means the selective ion permeation through the IEMs, has a large effect on the OCV [6]. Another major performance parameter to be considered is the stack resistance (*R_stack_*). The equation representing the *R_stack_* of RED is as follows:

(2) $$R_{stack}=\frac{n}{A}\left(R_{aem}+R_{cem}+\frac{d_{c}}{k_{c}}+\frac{d_{d}}{k_{d}}\right)+R_{el}$$

where *n* is the number of cell pairs, *A* is the effective area of the IEM (m^2^), *R_aem_* is the electrical resistance of the anion-exchange membrane (AEM, Ω m^2^), *R_cem_* is the electrical resistance of the cation-exchange membrane (CEM, Ω m^2^), *d_c_* is the thickness of the seawater compartment (m), *d_d_* is the thickness of the freshwater compartment (m), *k_c_* is the ion conductivity of seawater (S/m), *k_d_* is the ion conductivity of the fresh water (S/m), and *R_el_* is the Ohmic resistance of the electrodes and their compartments (Ω). From Equation (2), it can be confirmed that the electrical resistance of the IEMs is a key factor that has a great influence on the *R_stack_*. Meanwhile, the maximum power density (*W_max_*), which represents the overall power generation performance of RED, is expressed as a function of the OCV and *R_stack_*, as shown in equation (3):

(3) $$W_{\max}=\frac{{\left(V^{o}\right)}^{2}}{4R_{stack}}.$$

Overall, it can be seen that in order to improve the power generation performance of RED, it is necessary to increase the permselectivity of the IEMs and at the same time lower the electrical resistance. In addition, the IEMs for RED applications must be durable and, above all, inexpensive [7,8]. Meanwhile, most commercial IEMs are reinforced with polymer fabrics to improve the weak mechanical strength of the ion-exchange polymer. Since ion-exchange polymers contain functional groups with strong hydrophilicity, they generally possess high water content, weak mechanical strength, and poor dimensional stability. Some ionomers (e.g., Nafion) structured with a strong hydrophobic backbone do not require reinforcing in some cases, but reinforcing is a prerequisite for dimensional stability and long-term stable use of IEMs. For this reason, the thickness of the IEMs increases, which increases the membrane resistance, and the manufacturing process becomes complicated, which elevates the fabrication cost [9].

In addition, the importance of a large-scale energy storage system (ESS) is gradually increasing for the efficient use of eco-friendly renewable energy. Since most renewable energy sources possess intermittent production characteristics, there is a problem in that an imbalance between energy consumption and supply occurs. Therefore, in order to solve this problem, it is necessary to develop an appropriate ESS that can be integrated with renewable energy sources [10]. In particular, redox flow batteries (RFBs) are known as one of the most promising ESS [11]. The RFBs have a number of advantages: A very fast electrode reaction by dissolving the redox couples in a solution is possible and the system owns a flexible design by the capacity rating independent from the power rating [12,13]. Various redox couples, such as zinc/bromine, zinc/cerium, bromine/polysulphide, iron/chromium, and all-vanadium, have been employed as redox couples in RFBs [14]. Among various types of RFBs, all-vanadium redox flow battery (VRFB) utilizing vanadium redox couples is the most widely used for large-scale energy storage [10,14,15]. Particularly, VRFB has the advantages of relatively high energy efficiency and less cross-contamination of vanadium redox species of the two half-cell electrolytes [16,17]. Figure 1b shows the configuration and working principle of the VRFB system. The IEM that prevents the mixing of the anolyte and catholyte is one of the most important components influencing the charge-discharge performance and lifespan of an RFB system. The membranes used in the RFB systems should possess low electrical resistance, highly selective permeability to specific ions, small diffusion coefficient for solvents, excellent chemical and mechanical stabilities, and low production cost [18]. In the case of VRFB, currently, the perfluorocarbon-based Nafion membrane is the most widely utilized, but research on alternative membranes is being actively conducted due to the expensive membrane cost and significant vanadium crossover problem [19,20,21]. Moreover, recently, VRFBs employing AEMs with high cost-effectiveness and relatively low crossover of redox ion species have been actively researched [22,23,24].

Among the various types of membranes, a pore-filled membrane (PFM) made by filling a functional polymer into a chemically inert and porous substrate with excellent mechanical strength is considered to be promising for energy conversion applications [25,26]. In the traditional classification, the PFM is an intermediate form between a homogeneous membrane and a heterogeneous membrane, which has excellent chemical and physical stabilities and can be manufactured inexpensively, similar to a heterogeneous membrane. At the same time, it is very close to a homogeneous membrane in terms of the electrochemical characteristics [27,28,29]. In addition, undesirable excessive membrane swelling can be effectively prevented by the use of the mechanically strong porous substrate. Therefore, recent studies have been attempted to employ the PFMs in various electrochemical energy conversion processes, including RED and RFB [19,30,31,32,33].

Even though many research results on PFMs have been reported as described above, it is necessary to study the composition of the filled ionomer optimized for various types of application processes. Particularly, it is very important to improve durability along with ion conductivity for successful applications of the PFMs. From this point of view, in this study, a composite reinforced PFM filled with a novel anion-exchange polymer of a new structure that can simultaneously achieve a high cross-linking degree and ion-exchange capacity (IEC) was proposed. In more detail, a base membrane was prepared by filling a polyethylene (PE) porous support of about 25 μm thickness with monomers for introducing anion-exchange groups and monomers for copolymerization and cross-linking, followed by in situ polymerization and quaternization. Additional quaternary ammonium groups could also be formed at the same time as cross-linking through post-treatment. Ultimately, we have tried to fabricate thin-reinforced AEMs that do not cause excessive swelling by increasing the content of ion-exchange groups and at the same time increasing the degree of cross-linking. That is, by increasing the content of ion-exchange groups, it was attempted to promote the transport of counter ions (increase in ion conductivity) and simultaneously suppress excessive swelling to minimize the transport of unnecessary ionic species (increase in permselectivity and decrease in crossover rate). Various electrochemical properties and mechanical strength of the AEMs were systematically measured. The prepared AEMs were also applied to RED for energy production and VRFB for energy storage and the process performances were evaluated. In addition, it has confirmed the chemical stability of the prepared AEMs by measuring both the Fenton oxidation and vanadium oxidation stability. The results of this study are expected to provide important information for optimizing PFMs for various energy conversion processes and developing them for practical applications.

## 2. Materials and Methods

### 2.1. Materials

Vinyl benzene chloride (VBC) and 2-(dimethylamino)ethyl methacrylate (DMAEMA) were used as monomers for preparing the AEMs, *p*-xylylene dichloride (XDC) and divinylbenzene (DVB) as a cross-linking agent, and benzophenone (BP) as a photoinitiator, and all were purchased from Sigma-Aldrich (St. Louis, MO, USA). Moreover, 1-allyl-3-methylimidazolium bis(trifluoromethylsulfonyl)imide (Im-TFSI, Kanto Chemical Co., INC., Tokyo, Japan) was chosen and used as a monomer containing an ion-exchange group. All reagents were used without any purification. A porous PE film (Hipore, thickness = 25 μm, Asahi Kasei E-materials Corp., Tokyo, Japan) was used as a support for fabricating the reinforced AEMs. In addition, as a commercial membrane for the performance comparison, Neosepta AMX (Astom Corp., Tokyo, Japan) was selected and employed.

### 2.2. Fabrication of Reinforced AEMs

**Figure 2:**
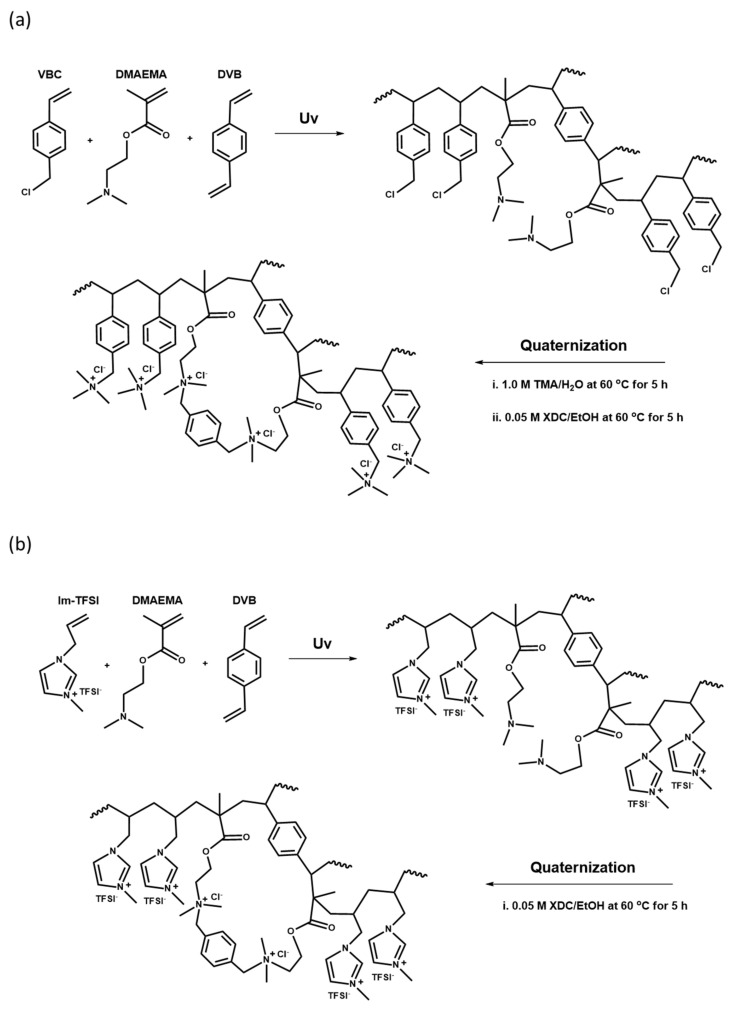
Reaction schemes of (**a**) quaternized poly(VBC/DMAEMA-DVB) and (**b**) quaternized poly(Im-TFSI/DMAEMA-DVB).

The base membranes were prepared with two mixed monomer compositions, VBC/DMAEMA and Im-TFSI/DMAEMA, and the molar ratio of the monomers was adjusted to VBC or Im-TFSI:DMAEMA=0.5~2.0:1. The contents of DVB used as a cross-linking agent and BP employed as a photoinitiator were 10 wt% and 3 wt%, respectively. The pore-filling was performed by immersing the PE porous support in the monomer mixture solution for 1 h. After that, the PE support filled with the monomer solution was placed in close contact between two sheets of release films and cured for 13 min using a high-pressure UV lamp (1 kW). Upon the completion of polymerization, the release films were removed and cross-linking and quaternization reactions were followed through post-treatments. In the case of VBC/DMAEMA, the base membrane was first immersed in 1.0 M trimethylamine (TMA) aqueous solution, and then the quaternization reaction was performed at 60 °C for 5 h to introduce quaternary ammonium groups in the VBC moiety. Thereafter, by reacting in a 0.05 M XDC solution in ethanol at 60 °C for 5 h, the cross-linking and the introduction of additional quaternary ammonium groups were simultaneously carried out. In the case of Im-TFSI/DMAEMA synthesized with a monomer containing an anion-exchange group, only the reaction in the XDC solution was performed under the same condition. After the post reactions, the membranes were washed with ethanol and distilled water, and then immersed in 0.5 M NaCl solution and stored. The membrane fabrication processes are schematically suggested in Figure 2.

### 2.3. Membrane Characterizations

Morphological characteristics of the surface and cross-section of the porous support and prepared AEMs were observed using a field emission scanning electron microscope (FE-SEM, TESCAN, Brno, Czech). In addition, the chemical structure of the fabricated membranes was analyzed using Fourier transform infrared spectroscopy (FT-IR, FT/IR-4700, Jasco, Tokyo, Japan). The water uptake (WU) of the commercial membrane and the prepared AEMs was calculated by measuring the difference between the wet weight (*W_dry_*) and the dry weight (*W_wet_*) of the membrane samples. The size of the membrane sample was 2 × 2 cm^2^, and after removing the moisture from the fully wetted sample surface using a filter paper, the wet weight was measured immediately. In addition, the dry membrane weight was measured after fully drying for more than 12 h in a drying oven at 80 °C. The measurement was carried out for a total of five samples, and the average weight values were used to calculate the WU values using the following equation [34]:

(4) $$WU=\frac{W_{wet}-W_{dry}}{W_{dry}}\times 100\;\;\left[\%\right].$$

Additionally, by measuring the wet volume (*V_wet_*) and dry volume (*V_dry_*) of the membrane, the volume swelling ratio (VSR) was calculated using the following equation [35]:

(5) $$VSR=\frac{V_{wet}-V_{dry}}{V_{dry}}\times 100\;\;\left[\%\right].$$

The IEC of the AEMs was determined by the Mohr method (titration). When the membrane sample reaches equilibrium in 0.5 M NaCl solution, it is washed with distilled water and then immersed in 0.25 M Na_2_SO_4_ solution for 6 h or more so that Cl^-^ ions in the membrane are completely replaced with SO_4_^2-^ ions. The amount of Cl^-^ in the substituted solution was quantitatively analyzed by the titration with a 0.01 M AgNO_3_ standard solution. In this case, K_2_CrO_4_ was used as an indicator. IEC values were calculated using the following equation [34]:

(6) $$\mathrm{IEC}=\frac{C\cdot V_{s}}{W_{dry}}\;\;\left[\frac{\mathrm{meq}.}{g_{\mathrm{dry}\;\mathrm{memb}}}\right]$$

where *C* is the normal concentration of the titration solution (meq./L), *V_s_* is the solution volume (L), and *W_dry_* is the weight of the dried membrane (g). Electrical resistance (ER) of the AEMs was evaluated in 0.5 M NaCl aqueous solution using a lab-made two-point probe clip cell and impedance analyzer (potentiostat/galvanostat, SP-150, Bio-Logic Science Instruments, Seyssinet-Pariset, France). The ER value was obtained through Equation (7) [36]:

(7) $$\mathrm{ER}=\left(R_{1}-R_{2}\right)\times A\;\;\left[\Omega \;{\mathrm{cm}}^{2}\right]$$

where *R*_1_ is the resistance of the electrolyte and the membrane (Ω), *R*_2_ is the resistance of the electrolyte (Ω), and *A* is the effective area of the membrane (cm^2^). The transport number indicating the selective transport of anions through the AEMs was measured by the traditional *emf* method using a two-compartment diffusion cell, and the calculation formula is as follows [37]:

(8) $$E_{m}=\frac{RT}{F}(2t_{+}-1)\ln\frac{C_{L}}{C_{H}}$$

where *E_m_* is the measured cell potential, *R* is the ideal gas constant, *T* is the absolute temperature, *F* is the Faraday constant, and *C_L_* and *C_H_* are the concentrations of NaCl solution, respectively, 1 mM and 5 mM. The cell potential was measured by connecting a pair of Ag/AgCl electrodes to a digital voltmeter. The mechanical strength of the commercial and prepared membranes was measured in a wet condition according to the international standard (ASTM D-882-79) using a universal testing machine (34SC-1, Instron, Norwood, MA, USA) [38]. Current-voltage (*I-V*) curves were obtained using a lab-made two-compartment cell equipped with a pair of Ag/AgCl plates and a pair of Ag/AgCl reference electrodes and filled with 0.025 M NaCl solution. The *I-V* responses were gained by connecting the Ag/AgCl electrodes to a potentiostat/galvanostat (SP-150, Bio-Logic Science Instruments, Seyssinet-Pariset, France). The permselectivity representing the selective ion transport through membranes in a RED process was measured using a two-compartment flowing cell. After placing an IEM between both compartments, seawater (0.513 M NaCl) and freshwater (0.017 M NaCl) were circulated at a flow rate of 50 mL/min, respectively. The membrane potential was measured for 30 min using a pair of Ag/AgCl reference electrodes, and then the average of the measured values was obtained. By substituting this result into Equation (9), the apparent permselectivity (*α*) in a RED process was calculated [39]:

(9) $$\alpha =\frac{\left[E_{m}/\left(\frac{RT}{F}\ln\frac{\alpha_{\pm }^{sL}}{\alpha_{\pm }^{sH}}\right)+1-2t_{M}^{s}\right]}{2t_{X}^{s}}$$

where *E_m_* is the potential difference of the IEMs, *R* is the ideal gas constant, *T* is the absolute temperature, *F* is the Faraday constant, $\alpha_{\pm }^{sL}$ is the average activity of ions in freshwater, $\alpha_{\pm }^{sH}$ is the average activity of ions in seawater, and $t_{M}^{s}$ and $t_{X}^{s}$ are the transport number of counter ions and coions in solution, respectively.

### 2.4. RED Performance Test

The RED performance employing different AEMs was measured in a galvanostatic mode by connecting a lab-made stack and a potentiostat/galvanostat (SP-150, Bio-Logic Science Instruments, France). Pt/Ti plates were employed as the electrodes, and the effective area of the electrodes and IEMs was 15 cm^2^, respectively. 0.05 M K_4_Fe(CN)_6_(II)/0.05 M K_3_Fe(CN)_6_(III) dissolved in 0.25 M Na_2_SO_4_ aqueous solution was used as an electrode compartment solution including redox couples. Concentrations of seawater and freshwater were 0.513 M NaCl and 0.017 M NaCl, respectively, and the solution volume was 100 mL each. The flow rate was 50 mL/min, and a 1 mm thick gasket made of polytetrafluoroethylene (PTFE) was used. The RED stack consisted of the total five-cell pairs and the current density was varied in the range of 0 to 14 A/m^2^ during the test.

### 2.5. VRFB Performance Test

For the evaluation of the charge-discharge performance of VRFB, a lab-made single cell was used. For the anolyte, 2.0 M V_2_(SO_4_)_3_/3.0 M H_2_SO_4_ aqueous solution was used, and for the catholyte, 2.0 M VOSO_4_/3.0 M H_2_SO_4_ aqueous solution was utilized. The volume of the anolyte and catholyte solutions was 16 mL, and the flow rate was 20 mL/min. Carbon felt (GF20-3, Nippon Graphite, Tokyo, Japan) was used as an electrode, and the effective area of the electrode and the membrane was 12.5 cm^2^, respectively. The carbon felt was used after successive heat treatments at 400 °C for 20 min and 500 °C for 10 min using a hot air blower for activation. The unit cell was charged up to 1.9 V and then discharged to 0.8 V using an automatic battery cycler (WBCS 3000, Wonatech Corp., Seoul, Korea) and the applied current density was 20 mA/cm^2^. To evaluate the charge-discharge performance of the VRFBs employing different AEMs, coulombic efficiency (CE), voltage efficiency (VE), and energy efficiency (EE) were calculated using the following equations, respectively [40]:

(10) $$\mathrm{CE}=\frac{\mathrm{Discharge}\;\mathrm{capacity}\;\left(\mathrm{Ah}\right)}{\mathrm{Charge}\;\mathrm{capacity}\;\left(\mathrm{Ah}\right)}\times 100\;\;\left[\%\right]$$

(11) $$\mathrm{VE}=\frac{\mathrm{Average}\;\mathrm{discharge}\;\mathrm{voltage}\;\left(V\right)}{\mathrm{Average}\;\mathrm{charge}\;\mathrm{voltage}\;\left(V\right)}\times 100\;\;\left[\%\right]$$

(12) $$\mathrm{EE}=\mathrm{CE}\times \mathrm{VE}\;\;\left[\%\right]$$

### 2.6. Overall Dialysis Coefficient of Vanadium Ion

The overall dialysis coefficient (*K_A_*) of vanadium cations through the membrane was calculated using a two-compartment cell (effective area = 4 × 4 cm^2^) filled with 1 M VOSO_4_/2.0 M H_2_SO_4_ (feed) and 1 M MgSO_4_/2.0 M H_2_SO_4_ (permeate). During the test, the solution absorbance was measured using UV/Vis spectroscopy (UV-2600, Shimadzu, Kyoto, Japan) to record the vanadium (VO^2+^) concentration in the permeate compartment over time. Finally, the *K_A_* value was determined using equation (13) [41]:

(13) $$K_{A}=\frac{k_{V}}{1+k_{V}}\frac{V^{II}}{At}\ln\frac{c_{A0}^{I}}{c_{A0}^{I}-\frac{1+k_{V}}{k_{V}}c_{A}^{II}}\;\;\left[\frac{m}{s}\right]$$

where $C_{A0}^{I}$ is the initial molar concentration of component A (i.e., VO^2+^) in the feed compartment, $C_{A}^{I}$ and $C_{A}^{II}$ are the molar concentration of component A in the feed (I) and permeate (II) compartments, respectively, *A* is the membrane effective area, $V^{I}$ and $V^{II}$ are the solutions volume in the feed (I) and permeate (II) compartments, respectively, *t* is time, and *k_v_* is the solution volume ratio of both compartments.

### 2.7. Chemical Stability Evaluation Tests

In order to confirm the oxidation stability of the commercial membrane and the prepared AEMs, the Fenton oxidation test was carried out. The membrane sample was prepared at a size of 2 × 2 cm^2^, impregnated in the Fenton solution prepared by mixing 3 wt% of H_2_O_2_ and 3 ppm of FeSO_4_, and maintained at 80 °C for 8 h [40]. The weight change was calculated by measuring the weights of the membrane at the initial stage and after the Fenton oxidation. In addition, a vanadium oxidation stability test was also performed to ensure the chemical stability of the AEMs in the VRFB system. This is based on the principle that VO_2_^+^ (V(V) species) is reduced to VO^2+^ (V(IV) species) by the oxidation reaction of a membrane immersed in a VO_2_^+^/H_2_SO_4_ solution [40]. For the measurement of vanadium oxidation stability, an AEM sample prepared in a constant size of 2 × 2 cm^2^ was immersed in 20.0 mL of 0.1 M (VO_2_)_2_SO_4_ (0.1 M V(V) in 5 M H_2_SO_4_) solution and maintained at 50 °C for 100 h [42]. The concentration of VO^2+^ ions in the solution was determined by measuring the solution absorbance using UV-visible spectroscopy (UV-2600, Shimadzu, Kyoto, Japan).

## 3. Results and Discussion

**Figure 3:**
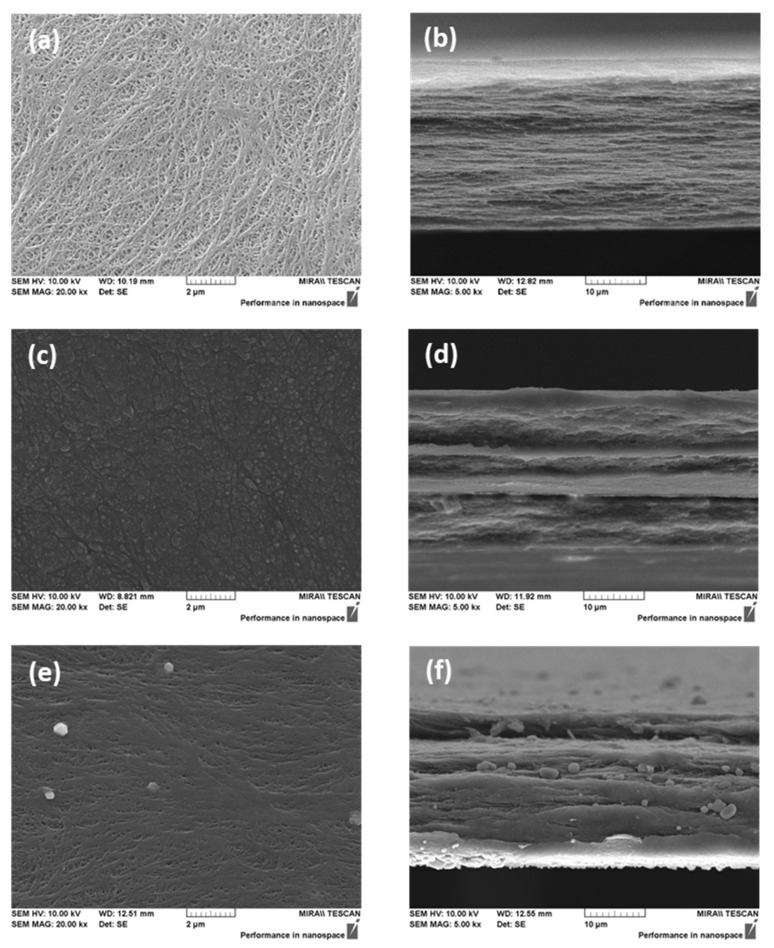
FE-SEM images of PE porous substrate ((**a**) surface; (**b**) cross-section) and pore-filled AEMs (VBC/DMAEMA ((**c**) surface; (**d**) cross-section) and Im-TFSI/DMAEMA ((**e**) surface; (**f**) cross-section).

Figure 3 shows the FE-SEM images of the porous substrate and the prepared AEMs. From the surface and cross-sectional images, open pores were not found in both types of the membranes (i.e., VBC/DMAEMA and Im-TFSI/DMAEMA), and therefore, it was believed that the pores of the porous support were completely filled with the ionomer.

**Figure 4:**
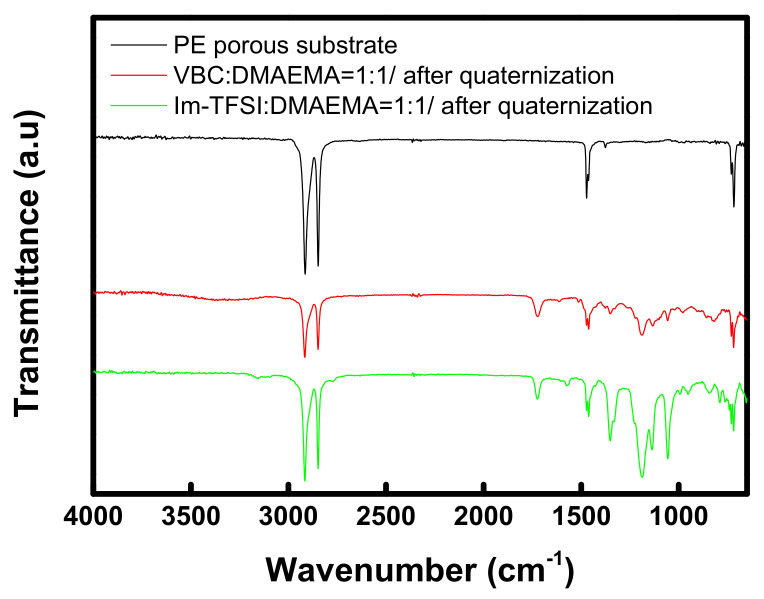
FT-IR spectra of PE porous substrate and pore-filled AEMs (VBC/DMAEMA and Im-TFSI/DMAEMA).

FT-IR analysis was performed to confirm the chemical structures of the prepared membranes, and the obtained spectra are summarized in Figure 4. For both VBC/DMAEMA and Im-TFSI/DMAEMA, an absorption band assigned to the C=O bond, implying the presence of DMAEMA moiety, was observed at 1722 cm^−1^, and an absorption band assigned to aromatic rings was found at 1462 cm^−1^ [43]. In addition, absorption bands corresponding to the C-N bond and quaternary ammonium groups were identified at 1195 cm^-1^ and 830 cm^-1^, respectively [44,45]. Meanwhile, in the spectrum of Im-TFSI/DMAEMA, it was found that the absorption band assigned to N=C-N stretching vibration of imidazolium group appeared at 1570 cm^-1^ [46]. As a result, it was confirmed that the ionomer having the chemical structure shown in Figure 2 was successfully filled in the PE porous support.

**Figure 5:**
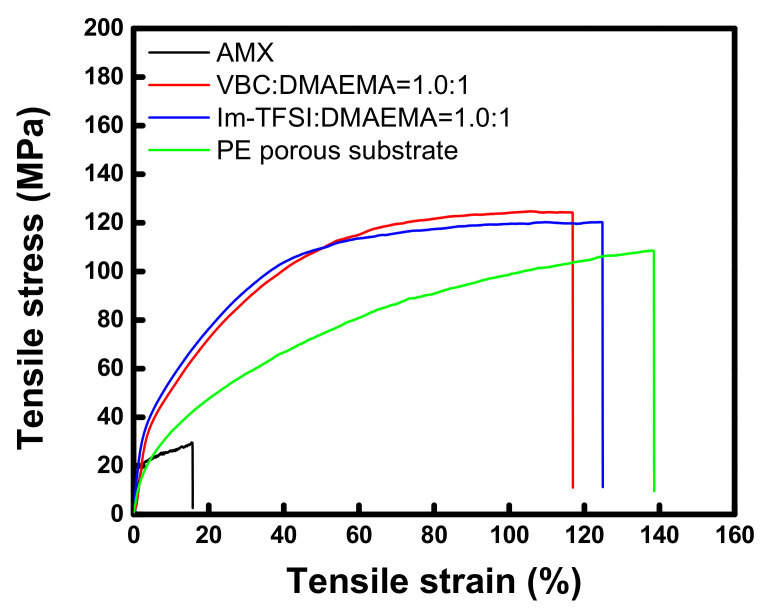
Tensile stress-strain curves of commercial membrane, PE porous substrate, and pore-filled AEMs (VBC/DMAEMA and Im-TFSI/DMAEMA).

The tensile stress-strain curves of the commercial membrane and the prepared AEMs, which are measured at a wet state, are displayed in Figure 5. The tensile strength and elongation at break values of the membranes are also summarized in Table 1. The tensile strength of the thin-reinforced AEMs is revealed to be about four times greater than that of the commercial membrane. From the results, it can be seen that the high toughness of the reinforced AEMs is originated from the strong mechanical properties of the PE porous film employed as the support. Meanwhile, it was observed that the tensile stress was increased while the strain was decreased by the successive pore-filling and in situ polymerization [47,48]. There was no significant difference in the physical properties of VBC-DMAEMA and Im-TFSI-DMAEMA, but the tensile strength of VBC/DMAEMA was slightly higher, whereas the elongation was higher in Im-TFSI/DMAEMA. This is considered to be related to the difference in the swelling degree and cross-linking density of the filled ionomers, that is, Im-TFSI/DMAEMA was expected to possess a higher swelling degree and lower cross-linking density than VBC/DMAEMA [49,50].

The basic characteristics of the commercial membrane and the prepared AEMs are summarized in Table 2. From the results, the IEC values of the thin-reinforced AEMs increased as the content of VBC or imidazolium monomer increased, and these were shown to be much higher than that of the commercial membrane. This is due to the additional quaternary ammonium groups generated in the process of cross-linking of DMAEMA moiety by XDC. Meanwhile, the VSR and WU of the prepared membranes showed a tendency to increase with the increase of the IEC. However, compared to the commercial membrane, it was found that the VSR and WU values were relatively low compared to the magnitude of the IEC, which is because the inert PE porous support used for membrane fabrication physically inhibits the excessive swelling of the ionomer [51]. In particular, VBC-DMAEMA appears to exhibit relatively low VSR and WU values as it has a relatively high cross-linking density and a dense structure compared to Im-TFSI/DMAEMA, as predicted from the mechanical properties. Im-TFSI/DMAEMA is considered to have a relatively loose structure because the polymerization occurs in a state containing bulky counter ions (i.e., TFSI anions) during the membrane fabrication. Note that TFSI anion has a molar volume about 3.6 times greater than that of Cl^-^ ion [52]. The ER of the membranes was also correlated with the IEC and showed a tendency to decrease as the IEC increased. Meanwhile, VBC/DMAEMA exhibits a relatively lower ion transfer resistance than Im-TFSI/DMAEMA, because it contained a large number of quaternary ammonium groups with higher polarity than the charge-delocalized imidazolium groups [53]. The transport number, which indicates the ion-selective permeability of the membrane, decreased as the VSR and WU values increased, but there was no significant difference among the membranes in the experimental range considered in this study. In general, the difference in transport number between membranes is not large within the range of appropriate IEC. Despite a small difference, the Im-TFSI/DMAEMA membranes showed lower transport numbers than the VBC-DMAEMA membranes, which could be related to the loose structure of the membranes.

**Figure 6:**
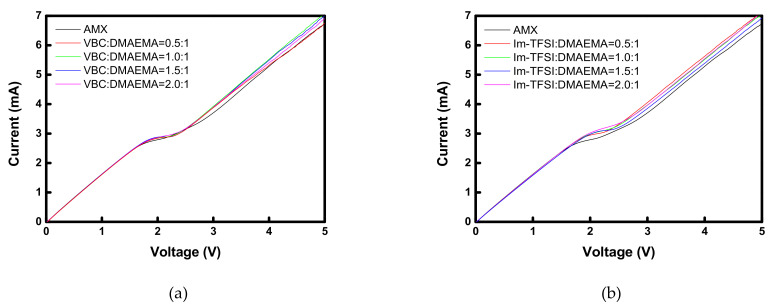
*I-V* curves of commercial and prepared membranes: (**a**) VBC/DMAEMA and (**b**) Im-TFSI/DMAEMA based pore-filled AEMs.

Figure 6 shows the *I-V* curves of the commercial membrane and the prepared AEMs. All the membranes showed typical three regions: an ohmic region, a plateau region demonstrating voltage rise due to concentration polarization, and a current increasing region due to electroconvection [38]. Therefore, it can be seen that the prepared membranes function properly as IEMs. In the case of VBC/DMAEMA, the *I-V* response was almost the same as that of the commercial membrane, but the Im-TFSI/DMAEMA membranes showed a somewhat higher limiting current density (LCD) value than that of the commercial membrane. This increase in the LCD value is a result of the decrease in the permselectivity and can be explained by the relatively loose structure of Im-TFSI/DMAEMA, as discussed previously [38].

**Figure 7:**
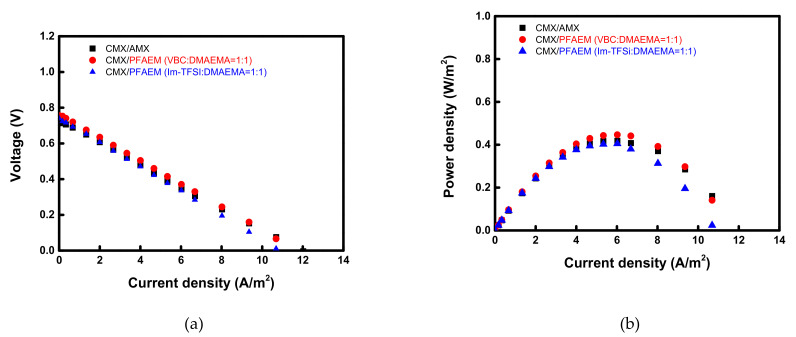
(**a**) Current-voltage and (**b**) current-power density curves of the electrochemical cells employing various AEMs.

Figure 7 exhibits the current-voltage and current-power curves of RED cells employing VBC:DMAEMA = 1:1 and Im-TFSI:DMAEMA = 1:1, respectively, which show the highest performance among the membranes fabricated for RED application. Here, the power density is displayed as values for one cell pair. The permselectivity and RED performance data of the tested AEMs are summarized in Table 3. It can be seen that VBC/DMAEMA exhibits higher power density compared to Im-TFSI/DMAEMA, which is a result of relatively high permselectivity and OCV, and low electrical resistance [54]. The VBC-DMAEMA membrane also showed a higher power density value than the commercial AMX membrane.

**Figure 8:**
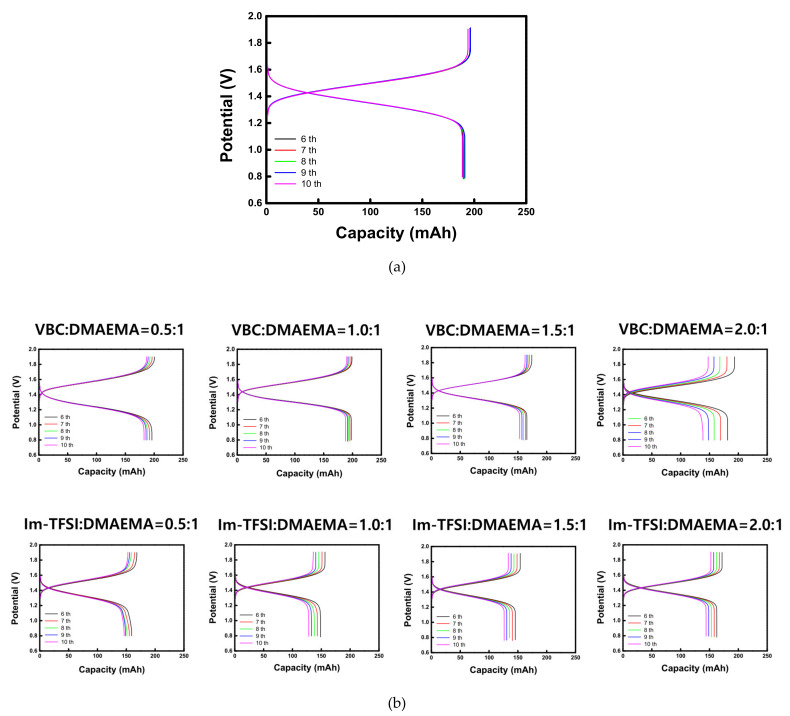
Charge-discharge curves of VRFBs utilizing (**a**) AMX and (**b**) prepared AEMs.

The charge-discharge performance of VRFB cells employing different AEMs was evaluated at 20 mA/cm^2^. The charge-discharge curves of the commercial membrane (AMX) and the prepared AEMs are shown in Figure 8. In addition, the battery performance parameters and the overall dialysis coefficients of vanadium ions are summarized in Table 4. For both VBC/DMAEMA and Im-TFSI/DMAEMA, as the IEC increased, the CE showed a tendency to decrease slightly, while the VE showed an increasing trend. The overall dialysis coefficients are the factors that indicate the degree of crossover of vanadium ions through the membrane. As the IEC increases, the membrane swelling elevates, which promotes the crossover of the vanadium ions, and thus, the CE decreases. In the case of the VE, it is greatly affected by the ER of the membrane, that is, as the IEC of the membrane increases, the ER decreases and the VE increases [40]. Since CE and VE have a trade-off relationship with each other, EE, which is determined by the product of the two efficiencies, could be optimized under specific conditions. As a result, VBC/DMAEMA showed the optimal performance at VBC:DMAEMA = 1:1 and Im-TFSI/DMAEMA at Im-TFSI:DMAEMA = 1.5:1. VBC-DMAEMA has lower vanadium crossover rate and ER compared to Im-TFSI/DMAEMA, demonstrating that it has relatively high EE. This result is considered to be because VBC/DMAEMA has a denser structure and stronger polarity than Im-TFSI/DMAEMA, as discussed above. As a result, the VBC-DMAEMA membranes showed superior charge-discharge performance than the commercial AMX membrane at the optimal composition. That is, in the case of VBC/DMAEMA = 1/1, it exhibited a relatively high CE because it showed a lower vanadium ion crossover, and also a high VE value due to a relatively low ER compared to the commercial membrane. Consequently, the EE value (87.4%) was increased by about 6.6% compared to the commercial membrane (80.8%). 

**Figure 9:**
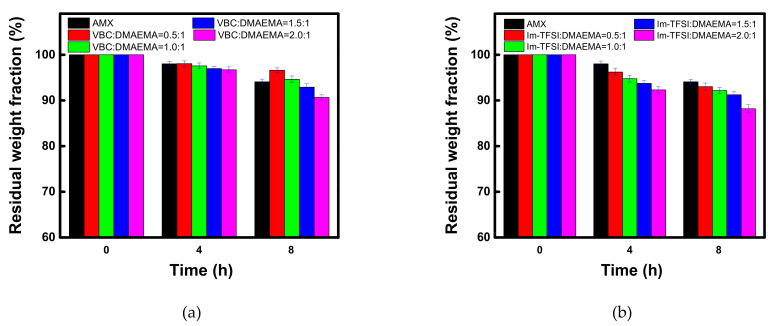
Time course changes in residual weight fraction during the Fenton oxidation test of (**a**) VBC/DMAEMA and (**b**) Im-TFSI/DMAEMA membranes.

The prepared membranes must have excellent durability for practical applications. Therefore, in this study, the chemical stability of the commercial membrane and the prepared AEMs was confirmed through the Fenton oxidation, as shown in Figure 9. The percent residual weight loss fraction values during the Fenton oxidation test are also summarized in Table 5. The decomposition by the Fenton oxidation reaction occurred significantly as the IEC increased for both VBC/DMAEMA and Im-TFSI/DMAEMA. The higher the IEC, the greater the polarity and the swelling degree of the membrane, which means that it could be easily attacked by radical species [55,56]. On the other hand, in the case of VBC/DMAEMA with a denser structure, at a molar ratio lower than VBC/DMAEMA = 1/1, the oxidation stability that was equal or higher than that of the commercial membrane, having a relatively small IEC, was exhibited. In addition, in the comparison of VBC/DMAEMA and Im-TFSI/DMAEMA, the relatively low oxidation stability of Im-TFSI/DMAEMA is thought to be due to easier access of radical species to ion-exchange groups and/or weak polymer chains in a bulkier structure [56].

**Figure 10:**
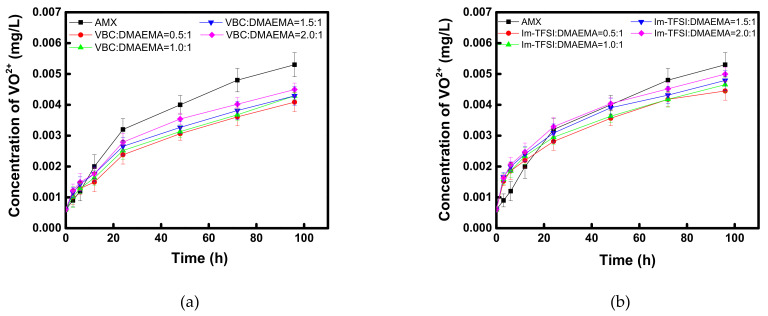
Time course changes in VO^2+^ concentration during the chemical stability test of (**a**) VBC/DMAEMA and (**b**) Im-TFSI/DMAEMA membranes in 0.1 M (VO_2_)_2_SO_4_/5 M H_2_SO_4_.

In addition, to evaluate the applicability of the prepared AEMs to VRFB, the oxidation stability under a practical electrolyte condition was checked as shown in Figure 10. In the case of VBC/DMAEMA, it showed superior vanadium oxidation stability compared to the commercial membrane, in all the considered compositions, and similar to the case of Fenton oxidation, it was found that the lower the content of the IEC, the higher the oxidative stability. Meanwhile, Im-TFSI/DMAEMA was inferior to VBC/DMAEMA but showed superior vanadium oxidation stability compared to the commercial membrane. From the results, it was considered that the movement and activity of the bulky vanadium ions are greatly affected by steric hindrance, as the polymer structure is denser [57]. Through the Fenton oxidation and vanadium oxidation tests, it was found that the reinforced AEMs developed in this study had excellent durability, equal to or greater than that of a commercial membrane, and thus, it was expected that the successful application to practical energy conversion processes would be possible.

## 4. Conclusions

In this study, thin-reinforced AEMs with high IEC and adequate swelling degree were prepared by filling VBC-DMAEMA or Im-TFSI/DMAEMA into a PE porous support, and through a double cross-linking reaction. The reinforced AEMs prepared by using a physically strong PE support exhibited excellent tensile strength that was improved four times or more compared to the commercial AMX membrane. Moreover, it was confirmed that the prepared membranes possess a lower ER than the commercial membrane due to the thin film thickness and high IEC. From the VSR and WU data, it was found that Im-TFSI/DMAEMA had a relatively bulky structure compared to VBC/DMAEMA, which was thought to be due to the inclusion of bulky TFSI anions during the polymerization. The commercial membrane and the prepared reinforced AEMs were applied to both RED and VRFB, which are promising energy conversion processes, and their performances were evaluated. From the results, it was confirmed that the performances of RED and VRFB were greatly affected by the swelling degree as well as the content and polarity of the ion-exchange groups of the AEMs. That is, VBC/DMAEMA with a denser structure and larger polarity showed relatively superior RED power density and VRFB charging-discharging performance compared to Im-TFSI/DMAEMA. It was also found that the reinforced AEMs exhibited superior energy conversion process performance compared to the commercial AMX membrane at the optimal composition (i.e., VBC:DMAEMA = 1:1). Meanwhile, as a result of evaluating the Fenton and vanadium oxidation stability of the membranes, the stability tends to decrease as the content of ion-exchange groups increases, and it can be seen that this is closely related to the swelling degree of the membrane. In conclusion, not only high performance of the energy conversion process but also excellent durability could be achieved by effectively suppressing the degree of swelling despite having a high IEC in the membrane.

## Figures and Tables

**Table 1:** The values of tensile strength and elongation at break of commercial membrane, PE porous substrate, and pore-filled AEMs (VBC/DMAEMA and Im-TFSI/DMAEMA).

Membrane	Tensile Strength (MPa)	Elongation at Break (%)
AMX (Astom Corp.)	29.47	15.60
VBC:DMAEMA=1.0:1/DVB0.10	124.3	116.9
Im-TFSI:DMAEMA=1.0:1/DVB0.10	120.2	124.8
PE porous substrate	108.5	138.4

**Table 2:** Basic properties of commercial membrane and prepared reinforced AEMs.

Membrane	Thickness (μm)	VSR (%)	WU (%)	IEC (meq./g)	ER (Ω·cm^2^)	Transport Number (-)
AMX	136	16.8	28.0	1.40	2.30	0.97
VBC:DMAEMA = 0.5:1/DVB0.10	24	9.67	17.0	1.95	1.25	0.97
VBC:DMAEMA = 1.0:1/DVB0.10	24	11.1	17.9	2.06	0.93	0.97
VBC:DMAEMA = 1.5:1/DVB0.10	24	11.7	18.1	2.16	0.86	0.96
VBC:DMAEMA = 2.0:1/DVB0.10	25	13.1	18.2	2.42	0.54	0.95
Im-TFSI:DMAEMA = 0.5:1/DVB0.10	24	16.7	26.9	1.74	1.81	0.96
Im-TFSI:DMAEMA = 1.0:1/DVB0.10	25	17.4	27.1	1.95	1.61	0.96
Im-TFSI:DMAEMA = 1.5:1/DVB0.10	25	18.0	29.2	2.08	1.40	0.96
Im-TFSI:DMAEMA = 2.0:1/DVB0.10	25	19.4	30.9	2.25	1.34	0.95

**Table 3:** Summary of membrane permselectivity and RED performance data of commercial and prepared membranes.

Membranes	Average Permselectivity, *α* (-)	OCV (V)	Power Density (W/m^2^, /Cell Pair)
CMX/AMX	0.831	0.732	0.418
CMX/VBC:DMAEMA = 1.0:1/DVB0.10	0.881	0.756	0.446
CMX/Im-TFSI:DMAEMA = 1.0:1/DVB0.10	0.872	0.740	0.404

**Table 4:** Summary of VRFB performance data of commercial and prepared membranes.

Membranes	CE (%)	VE (%)	EE (%)	$K_{VO^{2+}}$ (×10^−7^, m/s)
AMX	96.2	84.0	80.8	1.92
VBC:DMAEMA = 0.5:1/DVB0.10	98.0	85.6	83.9	1.74
VBC:DMAEMA = 1.0:1/DVB0.10	99.3	88.0	87.4	1.69
VBC:DMAEMA = 1.5:1/DVB0.10	97.3	88.5	86.1	1.89
VBC:DMAEMA = 2.0:1/DVB0.10	94.6	90.5	85.6	2.01
Im-TFSI:DMAEMA = 0.5:1/DVB0.10	95.6	78.0	74.6	1.84
Im-TFSI:DMAEMA = 1.0:1/DVB0.10	94.6	82.9	78.4	1.94
Im-TFSI:DMAEMA = 1.5:1/DVB0.10	94.3	85.6	80.7	2.02
Im-TFSI:DMAEMA = 2.0:1/DVB0.10	93.5	85.9	80.3	2.15

**Table 5:** Residual weight fraction during the Fenton oxidation test of commercial and prepared membranes.

Membranes	Residual Weight Fraction (%)	
	4 h	8 h
AMX	98.0	94.1
VBC:DMAEMA=0.5:1/DVB0.10	98.0	96.6
VBC:DMAEMA=1.0:1/DVB0.10	97.6	94.6
VBC:DMAEMA=1.5:1/DVB0.10	97.0	92.9
VBC:DMAEMA=2.0:1/DVB0.10	96.7	90.7
Im-TFSI:DMAEMA=0.5:1/DVB0.10	96.2	93.0
Im-TFSI:DMAEMA=1.0:1/DVB0.10	94.8	92.2
Im-TFSI:DMAEMA=1.5:1/DVB0.10	93.7	91.2
Im-TFSI:DMAEMA=2.0:1/DVB0.10	92.3	88.2

## Data Availability

Not applicable.
